# Efficacy and Safety of 4.7 mg Deslorelin Acetate Implants as a Neutering Option in Male Cats: A Large-Scale Multicentre Randomised Controlled Study

**DOI:** 10.3390/ani13030379

**Published:** 2023-01-22

**Authors:** Joana Amaral, Philippe Briantais, Christelle Fontaine, Delphine Rigaut

**Affiliations:** 1 Research & Development, Licensing Department, Virbac, 06515 Carros, France; 2 Global Marketing & Market Development Department, Virbac, 06515 Carros, France

**Keywords:** behavior, castration, contraception, deslorelin, implant, infertility, neutering, male cat

## Abstract

**Simple Summary:**

The control of feline fertility and sexual behavior has always been a necessity to facilitate cat cohabitation and relationships. Different methods are available for this purpose, with variable advantages. Beyond surgery, society’s consciousness of animal welfare has led to demands for less invasive methods of neutering that are easy to implement. This large field study aimed to confirm the interest in administering a deslorelin implant (Suprelorin^®^ 4.7 mg, Virbac, France) in tom cats. The results show that Suprelorin^®^ 4.7 mg suppresses fertility, sexual behavior and urine odor for at least 1 year when administered to male cats aged 3 months of age or older. Suprelorin^®^ 4.7 mg is therefore an effective and safe neutering option in male cats.

**Abstract:**

This multicenter-controlled, double-masked randomized European study was conduc-ted to confirm both the efficacy and safety of a deslorelin implant in controlling fertility and sexual behavior in a large population of tom cats over a 12-month period. Among the 225 screened individuals, a total of 205 privately owned indoor intact male cats, aged 3 months of age or older, were randomly allocated to a deslorelin implant (n = 154) or to a negative control group (n = 51). After the screening visit performed between day (D)-14 and D-7, six additional visits were sche-duled on D0, D45, D93, D186, D279 and D372. Effects on testosterone, sexual behaviors, penile spines, testicular volume and intact male cat urine odor were assessed at every visit under masked conditions as regards to the treatment group. In addition, phone calls from the investigators to the owners were scheduled on D7 and then on a monthly basis whenever no visit was scheduled. Success was defined as an individual serum testosterone concentration below or equal to 0.10 ng/mL and was 77.9% at D45, 83.1% at D93, 84.4% at D186 and D279, and 61.7% at D372 in the deslorelin group, and 3.9% at D45, 5.9% at D93, 3.9% at D186, 7.8% at D279 and 3.9% at D372 in the negative control group. Testing for superiority was made stepwise from D45 to D372 upwards; the difference in success rates was significant from D45 to D372 (*p* < 0.001 for each time point). The mean testosterone concentration dropped from baseline in the deslorelin group, remaining below the set threshold of 0.1 ng/mL until D372. From D7 onwards, the mean sum score for sexual behaviors (including vocalization, urine marking, aggression and intact male cat urine odor) was significantly lower at each observation time point in the deslorelin group compared to the control group, where no decrease in scores was observed. The mean percent change to baseline of the testicular volume and the percentage of cats with a decreased visibility and adult appearance of penile spines were significantly lower in the deslorelin group as soon as D45. No relevant safety concerns were reported during the course of the study. The deslorelin implant Suprelorin^®^ 4.7 mg (Virbac, Carros, France) is a safe and effective neutering option, inducing infertility over a 12-month period when administered to intact male cats aged between 3 months of age and 11 years of age. The implants also successfully reduced sexual behaviors (i.e., vocalization, urine marking, aggression), intact male cat urine odor, testicular volume and penile spine score for 1 year (372 ± 5 days).

## 1. Introduction

Controlling feline reproduction has been a long-time concern in managing populations and behaviors. In male cats, the most common neutering approach is gonadectomy [1]. In addition to the suppression of fertility, gonadectomy positively impacts sexual behaviors such as aggression, roaming and urine spraying [2]. Nevertheless, gonadectomy is not without potential detrimental effects on health, including surgical and anesthetic complications, overweight and diabetes mellitus [2]. 

Contraception products represent an alternative to surgical procedures. In male cats, progestins [3] can be used for the suppression of male sexual behavior, including urine spraying and improvement in intercat behavior. Nevertheless, fibroadenomatosis and the development of mammary adenocarcinomas have been described in tom cats after repeated injections of medroxyprogesterone acetate [4]. Likewise, a case of gynecomastia resulting in the need for a radical mastectomy was caused by daily administration of cyproterone acetate (5 mg/day) in a male cat [5]. Moreover, progestins are not reported to be effective for reproductive indications.

Intratesticular injections of sclerosing agents such as calcium chloride or zinc gluconate can also induce delayed prolonged infertility in adult male cats [4]. This method could cause permanent infertility and therefore is not indicated for temporary control of fertility.

Gonadotrophin-releasing hormone (GnRH) plays an important role in the reproductive function of mammals. This decapeptide, synthesized in the neurons of the hypothalamus, is secreted in a pulse-like manner into the hypothalamohypophyseal portal system. Upon reaching the pituitary gland, GnRH stimulates the secretion of the follicle-stimulating hormone (FSH) and luteinising hormone (LH) into the bloodstream [6]. In males, LH is mainly responsible for testosterone secretion by the Leydig cells. Testosterone together with FSH acts on the seminiferous tubules to stimulate Sertoli cells, which support spermatogenesis [7]. As a consequence, GnRH has been used as a target for immunocontraception, and GnRH vaccines have proven to be successful in the long-term suppression of fertility in cats [4]. In one study, 93%, 73%, 53% and 40% of the vaccinated cats remained infertile over the first, second, third and fourth years following vaccination, respectively [4]. However, vaccination induced persistent granulomatous injection site masses in five out of the 15 vaccinated cats for 2 years. The need for a booster injection and the unpredictable outcome, with a mean duration of efficacy following a single injection varying between 5 months and more than 5 years, makes its use challenging [4] in practice.

GnRH antagonists have also been actively studied. They present an immediate effect of suppressing the reproductive hormones, but the high-dose requirement in turn prevents their formulation into long-term releasing technologies. The first generation of antagonists has been associated with histamine release reactions, and the newer third and fourth generations still have limited effective durations [2]. 

Suprelorin^®^ (Virbac, Carros, France) is composed of deslorelin, a highly potent GnRH agonist [8]. Deslorelin’s binding affinity for the GnRH receptor (GnRH-R) is higher than that of endogenous GnRH. Additionally, it presents less susceptibility to enzymatic cleavage and therefore has increased potency. The continuous slow release of deslorelin from the Suprelorin^®^ implant induces a sustained stimulation of the GnRH receptors and enhances a complex network of transduction pathways involved in gene expression, resulting in an inhibition of the mRNA coding for the β-subunits of the gonadotrophins while increasing the free α-subunit levels in the serum. The whole mechanism of action of GnRH and consequently that of its agonists is not fully understood yet, but it also seems to be able to regulate the expression of its own receptor [9]. 

Suprelorin^®^ is licensed in the European Union for the induction of temporary infertility in adult male dogs and ferrets, and has been available for male cats since 2022. Published articles on small populations have demonstrated that slow-release 4.7 mg deslorelin implants showed variable individual results in testosterone secretion. Goericke-Pesch et al. found the latter to be initially stimulated, though non-significantly, and then downregulated from 3 to 11 weeks after implantation [10]. The time to downregulation is not correlated with the body weight [10]. As a consequence of the basal testosterone (<0.1 ng/mL), azoospermia can also be found in tom cats [11,12], and is likely to be maintained until 5 to 7 months after implantation [11]. Nevertheless, the underlying testicular histology seems variable, showing all developmental levels of spermatogenesis [3,11,12,13]. The mean duration of “clinical efficacy” has been described to be around 18 months, with the disappearance of sexual behaviors and other testosterone-related behaviors such as urine marking, vocalization or aggression [14]. Nevertheless, there is high individual variability in the evolution of these different parameters [15].

This study, which supports the official registration of Suprelorin^®^ 4.7 mg for male cats, aimed to confirm these results in a large-scale field study.

## 2. Materials and Methods

### 2.1. Animals

This double-masked, negative-controlled, randomized and multicentre study was conducted in 3 European countries (Germany, Hungary and Portugal) in 16 different veterinary clinics, according to their European and national regulatory requirements. Ethical approval was given by the relevant ethical committee with the registration number 18–14.

A total of 205 privately owned healthy, intact and indoor male cats of 3 months of age or older, with normal genital appearance and both testicles descended in the scrotum, from a single or multi-cat household were enrolled in the study. Excluded from enrolment were outdoor and/or cryptorchid cats, cats who had been under hormonal or glucocorticosteroid therapy since birth, cats with any concomitant disease that would interfere with the evaluation of the study treatment (including but not limited to neoplasias, FIV/FeLV, phimosis/paraphimosis, orchitis, epididymitis and testicular trauma), cats diagnosed with a new disease at the screening or inclusion visit and cats with an already known concomitant disease that was not stabilized and controlled at the screening visit. 

Some animals were removed and discontinued the study prematurely if requested by the owner or in the event of non-compliance, adverse effects, death, euthanasia or concomitant disease requiring non-permitted drugs (sexual hormonal treatments or glucocorticosteroids).

### 2.2. Study Design

Included cats were randomly allocated to a deslorelin group (Suprelorin^®^ 4.7 mg, VIRBAC, Carros, France, n = 154) or to a negative control group (1 mL of 0.9% sodium chloride, n = 51). Both the deslorelin implant and sodium chloride solution were injected subcutaneously between the shoulder blades. The administration of the investigational products was performed by a treatment administrator after enrolment on day (D)0.

### 2.3. Animal Follow-Up

After inclusion, five study visits were scheduled on D45 ± 3, D93 ± 5, D186 ± 5, D279 ± 5 and D372 ± 5. In addition, to ensure protocol compliance, phone calls from the veterinarians to the owners were performed on D7 ± 1 and then on a monthly basis whenever no visit was scheduled.

### 2.4. Evaluation Criteria

#### 2.4.1. Efficacy

Treatment success at each visit was defined as having a serum testosterone level ≤0.10 ng/mL between D45 and D372. Testosterone concentrations were assessed on the day of the screening (between D-14 and D-7), D45, D93, D186, D279 and D372 and the percentage of cats achieving treatment success was determined from D45 to D372.

Different sexual behaviors were assessed by the owners during the screening visit and then on D0, D7, D31, D62, D93, D124, D155, D186, D217, D248, D279, D310, D341 and D372, with an allowed time window of ±1 to ±5 days. A combined sexual behavior score was calculated as a sum score of vocalization, urine marking, aggression and intact male cat urine odor scores for each individual cat, at each time point. This aggregate score ranged from 0 to 8 (Table 1). The percent change from baseline was then calculated, and both groups were compared.

Assessments of penile spines and testicular volume were evaluated by the investigators during the screening visit and then on D45, D93, D186, D279 and D372. Penile spines were evaluated by visual inspection on a 0–2 scale (0: no penile spines visible, 1: penile spines just visible, 2: penile spines with adult appearance, clearly visible, large and pointed). Individual penile spines scores and their change from baseline were calculated for each time point. The testicular length, height and width of the left and the right testicles were measured using a calibrated calliper. The volume of each testis was calculated according to the formula volume (mm^3^) = 4/3 * π * (1/2 *a* * 1/2 *b* * 1/2 *c*), where “*a*” is the testicular length (mm), “*b*” is the testicular height (mm) and “*c*” is the testicular width (mm). The testicular volume for each cat was defined as the mean volume of the right and left testis at each time point. The percent change from baseline was calculated and compared between groups.

#### 2.4.2. Safety 

Safety was monitored regularly throughout the study. Physical examinations were performed each visit. Based on our knowledge of deslorelin’s mode of action and previous safety results for cats, and to limit the amount of blood sampling procedures during visits, haematology and blood biochemistry analyses were performed at four time points during the study, including on the day of screening and on D45, D186 and D372. Urine analysis was performed at the screening visit and at the end of the study on D372. Implantation site reactions were monitored by both owners and investigators. Furthermore, any abnormal general clinical signs, changes in body weight and appetite and abnormal testicular evaluation results were reported on the clinical form. 

### 2.5. Data Analysis

Efficacy and safety were assessed in all randomized animals that received at least one dose of either deslorelin or the sodium chloride. Efficacy analyses were based on the intention-to-treat (ITT) population using the last-observation-carried-forward method for handling missing data. The ITT population matched the safety population. Results are presented as mean ± standard error (SE).

All analyses were performed using SAS version 9.4 software. A two-sided 5% significance level was used. Efficacy criteria were compared between groups at each time point between D45 and D372 using the Cochran-Mantel-Haenszel test stratified by center with the RIDIT transformation and the general association statistic for binary endpoints and row mean score for continuous endpoints (FREQ procedure from SAS).

## 3. Results

### 3.1. Study Population

A total of 225 intact male cats were initially screened. Among them, 20 cats were considered not eligible for inclusion. Thus, 205 were randomly allocated to the deslorelin and the control groups, (154 and 51 cats, respectively). The mean age of the cats was 1.63 ± 0.159 years in the deslorelin group and 1.47 ± 0.248 years in the control group. The mean body weight was 3.78 ± 0.087 kg in the deslorelin group and 3.77 ± 0.145 kg in the control group. The majority of enrolled cats were crossbred (89.61% and 86.27% in the deslorelin and control groups, respectively).

Of the 205 enrolled cats, a total of 141 (68.8%) animals (128 (83.1%) in the deslorelin group and 13 (25.5%) in the control group) completed the study on D372. Altogether, 64 (31.2%) animals (26 in the deslorelin group and 38 in the control group) were prematurely withdrawn from the study. Removals were mainly due to a perceived lack of efficacy by owners (four animals in the deslorelin group and 29 animals in the control group) and the non-compliance of the owners (six cats in the deslorelin group and four cats in the control group). A total of 10 cats were removed for “other reasons” (eight in the deslorelin group and two in the control group, mainly due to cat disappearance or domestic accidents). Five additional animals were withdrawn because of suspected adverse effects or concomitant disease (two and three in the deslorelin and control groups, respectively). Five cats from the deslorelin group were euthanized or died due to causes unrelated to the condition under investigation. Finally, one cat from the deslorelin group was removed due to protocol non-compliance. 

### 3.2. Testosterone Concentration

In the deslorelin group, the mean testosterone concentration dropped to values below the set threshold of 0.1 ng/mL from D45 onward. Testing for superiority was performed stepwise from D45 to D372 onward. Significance in the difference in success rates between the deslorelin group and the control group was reached at D45 and maintained until D372 (*p* < 0.001).

**Figure 1:**
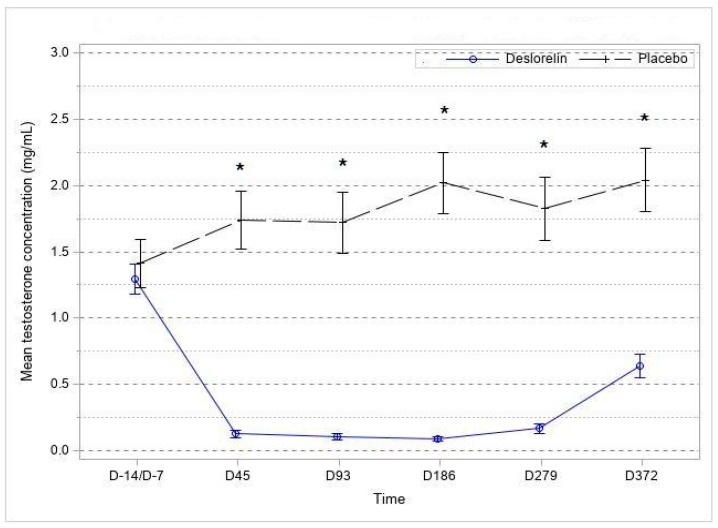
Evolution of serum testosterone level over time (expressed as mean ± SE). * Significant difference (*p* < 0.001) between groups from D7 onward.

The percentage of cats with serum testosterone below or equal to 0.1 ng/mL was 77.92% at D45, 83.12% at D93, 84.42% at D186 and D279 and 61.69% at D372 in the deslorelin group and 3.9% at D45, 5.88% at D93, 3.92% at D186, 7.84% at D279 and 3.92% at D372 in the control group. For approximately 90% of the cats in the deslorelin group, the mean serum testosterone levels remained below 0.2 ng/mL from D45 to D279 (D45: 92.2%, D93: 93.5%, D186: 93.5%, D279: 88.3%) and went above 0.5 ng/mL on D372. The average testosterone concentration in the control group remained above 1.5 ng/mL from D45 to the end of the follow-up (Figure 1).

Almost all deslorelin-treated cats had very significantly reduced testosterone levels throughout the study compared to baseline and control cats, and only at the last time point (D372) did some treated cats start to show a reverse to pre-treatment testosterone levels.

### 3.3. Sexual BehaviorSum Score

**Figure 2:**
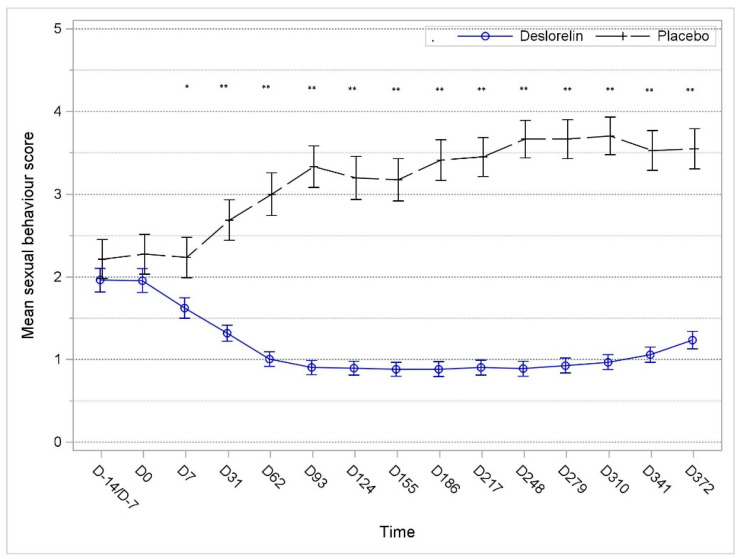
Course of mean sexual behavior score (expressed as mean ± SE) in the deslorelin and control groups over time. * Significant difference (*p* = 0.013) between groups. ** Significant difference from D31 onward (*p* < 0.001).

The mean sexual behavior score (ranging from 0 to 8) decreased and remained lower compared to that on D0 throughout the study period in the deslorelin group, whereas it substantially increased in the control group. The distribution of the sexual behavior score significantly decreased as soon as D7 (*p* = 0.013) and again at each additional observation time point in the deslorelin group compared to the control group (*p* < 0.001) (Figure 2).

### 3.4. Sexual Behavior Individual Scores

#### 3.4.1. Vocalization

In the deslorelin group, the vocalization score steadily decreased after treatment, whereas no change was recorded in the control group. The change from baseline between groups was significantly higher for the deslorelin group at each post-baseline observation time point after D0 (*p* < 0.001).

#### 3.4.2. Urine Marking and Urine Odor

**Figure 3:**
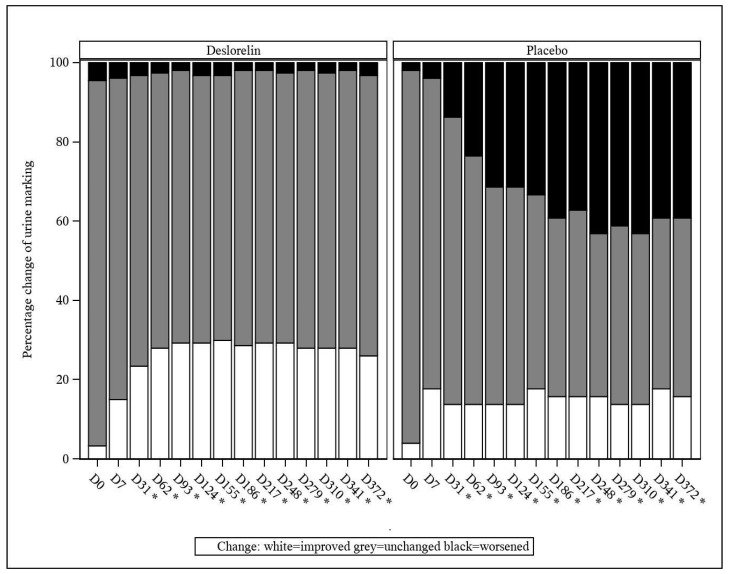
Percentage change in urine marking compared to baseline. * Significant difference (*p* < 0.001) between groups from D31 onwards.

**Figure 4:**
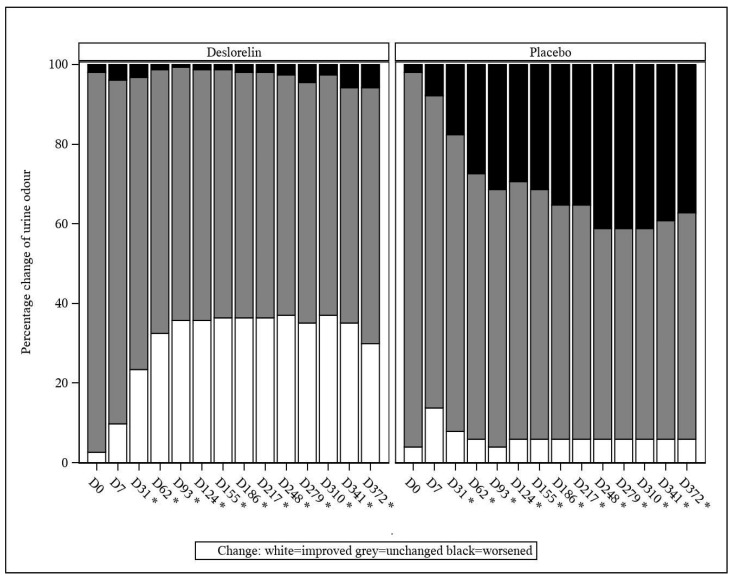
Intact male cat urine odor percentage change compared to baseline. * Significant difference (*p* < 0.001) between groups from D31 onwards.

From D31 onwards, a decrease in the incidence of urine marking (classified as an improvement) was observed at a significantly higher percentage in the deslorelin group than in the control group (*p* < 0.001; Figure 3). The same occurred from D31 onwards, with worsening of the intact male cat urine odor being observed at a significantly lower percentage in the deslorelin group compared to the control group (*p* < 0.001; Figure 4).

#### 3.4.3. Aggression

The score for aggression (ranging from 0 to 3) gradually decreased from baseline in the deslorelin group, whereas no decrease was observed in the control group. Changes from baseline became significantly different between groups as soon as D31 (*p* ≤ 0.005).

### 3.5. Penile Spines

In the deslorelin group, from D45 onwards, the adult appearance of penile spines was observed in a lower percentage than in the control group. The difference between groups in regard to the decrease in penile spine score from baseline was statistically significant at each visit from Day 45 onwards (*p* < 0.001).

### 3.6. Testicular Volume

The mean testicular volume (mean of left and right testes) was lower from D45 onwards in the deslorelin group compared to baseline, whereas it continuously increased in the control group throughout the study period. The mean percent changes in testicular volume compared to baseline were significantly lower in the deslorelin group than in the control group from D45 to D372 (*p* < 0.001).

### 3.7. Safety

According to the owners´ assessments, at least one implantation site reaction (including redness, pain or heat) was observed in five (3.25%) animals in the deslorelin group, whereas no reactions were reported in the control group, with a significant difference between groups. Based on the physical examinations carried out by the investigator at each visit, nine (5.84%) animals in the deslorelin group and one animal (1.96%) in the control group exhibited a single local reaction, with no statistical differences between groups. Slight swelling (<5 mm) was reported occasionally in less than 3% of the animals up to D45, with no significant differences between groups (according to confidence intervals including the null value). 

No statistical differences were observed between groups regarding the number of animals that experienced at least one adverse event (AE) or at least one serious AE. One cat in the deslorelin group developed a mass post-implantation on D76 located at the left thoracic wall, separate from the implantation site, which was set between the shoulder blades. After its surgical removal, histopathology diagnosed a fibrosarcoma. No implant remnants were found in the histopathological report of the tumoral mass. As there was no association with the location and no fragments of the implant, the relationship with the implant was assessed as unclassifiable.

Over the course of the study, no relevant differences were noticed between groups regarding general clinical examinations. One animal, in the deslorelin group, showed soft testicles on D45 and D93. 

The mean body weight increased by 0.75 kg in the deslorelin group versus 0.35 kg in the control group. Weight gain was statistically higher in the deslorelin group on D186 and D372 (*p* = 0.0323 and *p* = 0.0003, respectively) when compared to the control group. Regarding appetite evaluation by the owner, the difference between groups was found to be statistically significant on D93 (*p* = 0.004), whereas the percentage of increased appetite was significantly higher in the deslorelin group than in the control group. No differences were seen at the other time points.

## 4. Discussion

This large-scale controlled field study screened a total of 225 intact male cats. Altogether, 205 were considered eligible for inclusion and were randomly allocated to the deslorelin group or to the negative control group (154 and 51 cats, respectively). The main efficacy criterion, defined as the percentage of animals that achieved levels of testosterone ≤0.1 ng/mL at every time point, was present as soon as D45 in 77.92% of cats in the deslorelin group (vs. 3.9% in the control group). The percentage of cats with testosterone ≤ 0.1 ng/mL was significantly higher in the deslorelin group compared to the control group from D45 to the end of the study period. The 0.1 ng/mL threshold for testosterone was based on previous publications [6,10] where the efficacy of the implant was demonstrated by a sustained decrease in testosterone concentrations to basal levels or levels below 0.1 ng/mL. When determining a male cat’s fertility, we should consider not only testosterone concentration but also parameters such as libido, mounting behavior, intromission, pelvic thrust, normal mobility of the penis and sperm quality [7]. 

In the literature, when testosterone measurements are performed before D45, a drop in testosterone concentrations is reported around 20 days after implantation [15]. One study conducted with adult male cats aged between 1 and 6 years reported a level of testosterone lower than 0.1 ng/mL in 50 percent of the cats 28 days after administration of a deslorelin implant and in all but one cat after 77 days [10]. 

As early as D7, the combined sum score for sexual behaviors was significantly lower at each post-baseline observation time point in implanted cats (*p* = 0.013 at D7, *p* < 0.001 for each visit from D31). Though slightly increased from D279, the combined sexual behavior score was still almost three times lower than that of the control group on D372, having remained stable below baseline values from D7 onwards. 

Urine marking, intact male cat urine odor and aggression were significantly lower in the deslorelin group than in the control group as soon as D31. A significant (*p* < 0.001) decrease in vocalization was also noticed as early as one week after implantation in the deslorelin group. In the literature, some of these unwanted sexual behaviors have been suppressed even longer in some implanted cats with a reported mean duration of 13.4 ± 3.2 months and a range of 8 to 21 months [16]. Interestingly, in our study, none of the behavior scores (vocalization, urine marking, urine odor and aggression) increased after the administration of the implant, though this initial stimulation has been described in several publications [10,17,18]. The flare-up effect has also been demonstrated in dogs, generally occurring a few days after implantation [18], being due to the agonist effect of deslorelin, which first increases FSH and LH secretion and subsequently sex steroid hormones. It generally ceases gradually. The possible interindividual variations and the fact that our first testosterone measurement was performed at 45 days post-implantation may have attenuated this observation from a statistical standpoint. On the other hand, there were no reports of behavioral changes from the day of treatment onwards reported by the owner that could have been attributed to the flare-up effect. It would be interesting to collect more data on the effects of the 4.7 mg deslorelin implant administered to prepubertal male cats, as one study found a 4.7 mg deslorelin implant administered from 3 months of age prevented any male-sexual-related behavior for 48 weeks [19]. In our study, 3 prepubertal male cats less than 4 months old were included in the deslorelin and in the placebo groups, respectively. The three cats administered the Suprelorin^®^ implant demonstrated hardly any detectable sexual male behavior. However, such a small sample size did not allow us to evidence any significance.

The decrease in testicular volume observed seems to slightly vary among publications. In a study from Goericke-Pesch et al., the testicular volume significantly decreased by about 60% on week 12 and about 73% after week 36 [10]. In a second study published in 2013, the mean testicular volume first increased in week 1 after implantation and then decreased by 50% in week 12 [13]. After 20 weeks, it decreased by 60% compared with the initial values. In a 2015 publication, Favre et al. reported a 60% volume reduction after 28 weeks of implantation [20]. In our study, the mean percent change in the testicular volume of the treated group compared to baseline was significantly lower than in the control group. (We included animals 3 months of age or older; 42% were younger than 9 months and 58% were older than 9 months at the time of enrolment, and equally distributed between groups.) 

From D45 onwards, a significantly (*p* < 0.001) higher percentage of implanted cats showed a decrease in penile spine scores than those in the control group. 

Secondary efficacy results support the efficacy of the deslorelin implant up to 12 months post-implantation. The current literature even describes efficacy up to 18.3 ± 3.0 months (range 15–25 months) [3] or 26.5 ± 7.42 months [21] (regarding basal testosterone and dihydrotestosterone suppression, respectively). This study was conducted and designed to support a minimum efficacy duration of 12 months. Longer follow-ups will require the implementation of larger and more complex studies. The analysis of the sexual secondary parameters implied that a testosterone level above 0.1 ng/mL should not be the only criterion for fertility assessment and should be assessed in combination with the appearance of any other sexual characteristics.

Six cats had high testosterone despite measurable deslorelin exposure, but still showed clinical suppression (four showed a partial clinical response and the other two demonstrated a full clinical response). Consequently, only four cats had an unsatisfactory response with regard to the testosterone levels at each time point in the study, despite measurable deslorelin exposure and a partial clinical response. This could be explained by biological variability in receptors or receptor affinity, or other sources of testosterone (e.g., adrenal glands), but this research field has been not developed for cats. Data found in the literature also report either a delay in or absence of testosterone downregulation. In experimental conditions, Goericke-Pesh et al. described one cat out of 10 reaching basal testosterone levels at 11 weeks post-implantation [10]. In another similar study [13], one cat out of five did not show basal testosterone over the 20-week follow-up. The proportion of cats with failed treatment regarding testosterone downregulation in our study does not appear to be unusual in comparison to other trials. Whatever the considered parameter (testosterone or semen quality), most trials conducted on a limited population described individual variation, complicating direct comparison [10,12].

There was no statistical difference between the groups regarding adverse events reported during the study. Amongst several studies and publications investigating the use of deslorelin in male cats, this was the largest study conducted on client-owned cats with a treated versus negative control group. The results found in this study are consistent with data published in the literature.

As this was a field study performed in client-owned cats, semen collections and semen quality—which is considered a main criterion of evidence of fertility in follow-ups—could not be investigated. Semen collection in cats requires electro-ejaculation, catheterizations under general anesthesia or well-trained animals, which is not possible in general day-to-day practice. Moreover, in contrast to male dogs, the effects on spermatogenesis on tom cats are highly variable, with the arrest of spermatogenesis being possible at various different stages of sperm development [13]. This interindividual variations [12] make this criterion difficult to implement when evaluating the fertility status of felines under field conditions.

The high dropout rate seen in our control group (59%) was related to the display of unwanted sexual behaviors such as aggression, spraying and vocalization, as opposed to only 2.6% in the implanted group. These data corroborate the discomfort these behaviors bring to a cat household, especially those with indoor cats. In a market survey led in 2017 by Harris, among pet owners [22], improving cats’ behavior was the main reason for choosing castration, especially in Europe (more than one responder out of three). Suprelorin^®^ has been shown to be very effective in reducing and even eliminating some of these behaviors. 

At the same time, based on some ethical concerns, it is argued that routine surgical neutering, at least in the case of non-free-ranging companion animals, raises significant ethical questions, and from some ethical perspectives, including those which posit companion animals have a right to bodily integrity and to not be harmed, is highly problematic [23]. This view is already supported by a few national laws [2]. 

The ease of administration, efficacy, safety and reversibility of the deslorelin implant explains why the implant is perceived as a promising alternative, non-surgical method for controlling male and female domestic cat reproduction, in particular by the owners of cats temporarily not intended for breeding. It is also recommended by veterinary practitioners for cats at increased anesthetic risk and by organizations in areas without easy access to a surgical facility [24].

Finally, though not studied in our experiment, published data support that deslorelin’s effects are reversible [24,25] and can even be shortened with the removal of the implant [15,26]. The restoration of sexual parameters, including testicular function and the reestablishment of spermatogenesis, has been confirmed in several cats after the removal of the implant [15]. Moreover, a return to normal reproductive function has also been demonstrated by the use of questionnaires. One questionnaire was sent to French cat breeders to evaluate the duration of effect, fertility after use, changes in behavior, etc. The survey presented data from 57 tom cats and 15 different breeds, treated 1–3 times. A total of 24 tom cats, after a full inhibition period, were subsequently presented to females, and all 24 (100%) mated successfully at a mean of 15.5 ± 3.5 months after implantation, leading to litters of 2–7 kittens [16]. Another questionnaire conducted as an online survey to cat owners from 28 countries who had used deslorelin implants in their tom cats with single or repeated administrations evaluated a total of 486 answers and 31 different breeds. In this survey, all the efficacy parameters were described to have reverted after the end of the implant period; 70.8% of the male cats had offspring before the implant, and no matter the number of implants they had been administered, 89.2% of the cats had offspring after the end of effectiveness of the drug [27]. 

To the authors’ knowledge, this is the largest prospective field study conducted in male cats with deslorelin acetate implants. Complementary data on the repeated administration of deslorelin implants in male cats would be valuable to further support their use in practice.

## 5. Conclusions

In this multicentre, double-masked, controlled randomized study, the administration of a 4.7 mg deslorelin implant to male cats aged 3 months of age or older significantly reduced the mean testosterone concentration, which was maintained below 0.1 ng/mL. From D7 onwards, the mean sum score for sexual behaviors (including vocalization, urine marking, aggression and intact male cat urine odor) was significantly lower in the deslorelin group compared to the control. The mean percent change from the baseline of testicular volume and the percentage of cats with decreased visibility and adult appearance of penile spines were significantly lower in the deslorelin group as soon as D45. The effects of the 4.7 mg deslorelin implant were maintained until D372, with individual variability reported in relation to testosterone downregulation. No relevant safety concerns were reported.

The hormonal downregulation of male cats by means of long-term deslorelin exposure showed some interindividual variability that requires further research studies.

Suprelorin^®^ 4.7 mg was confirmed to be a reliable neutering option, providing a safe and effective reduction in fertility, sexual behavior and urine odor over a 12-month period when administered in male cats 3 months of age or older.

## Figures and Tables

**Table 1:** Scoring system used to describe the different sexual behaviors. The total combined sexual behavior score corresponds to the sum score of individual behaviors at every time point and is rated from 0 to 8.

Description	Method of Measurement
Vocalization, e.g., meowing, yowling, growling	0: Never observed 1: Observed occasionally (not more than 5 times a day and not more than two meows at a time) 2: Observed frequently (more than 5 times a day or more than two meows at a time) 3: Continual vocalization observed
Urine marking (spraying of urine (mostly on vertical surfaces, e.g., walls) with lifted, sometimes quivering, tail)	0: Not observed 1: Observed
Intact male cat urine odor (strong and pungent odor of cat and/or urine)	0: Not observed 1: Observed
Aggression towards humans or fighting (scratching, biting)	0: Never observed 1: Observed occasionally (not more than once a day) 2: Observed frequently (more than once a day) 3: Observed at every contact

## Data Availability

The data are not publicly available. Specific request for additional information can be sent to the corresponding author at christelle.speiser-fontaine@virbac.com.
